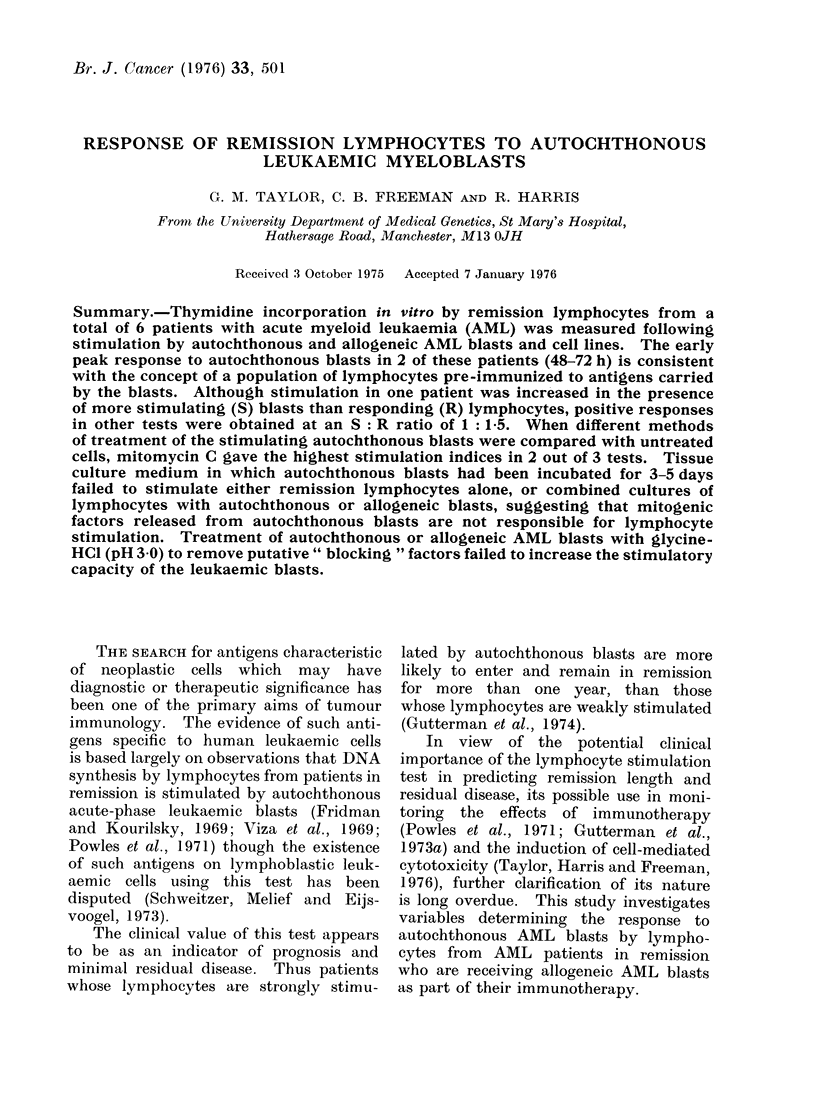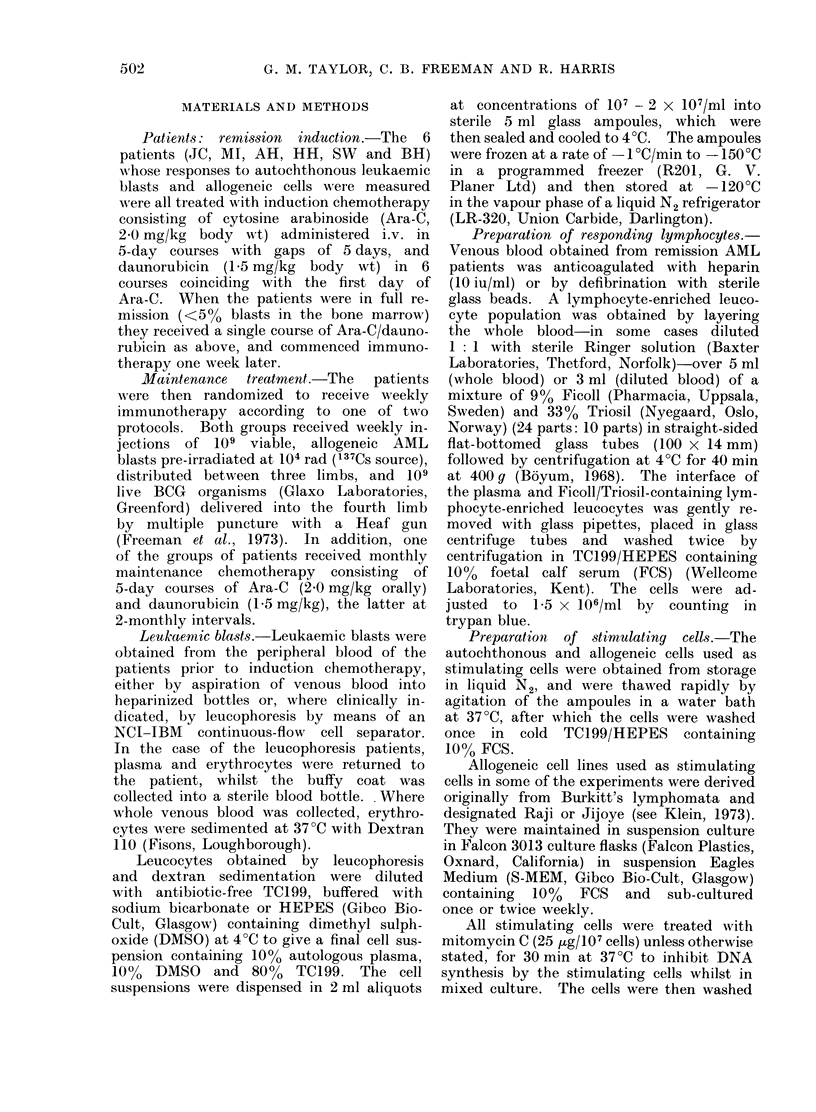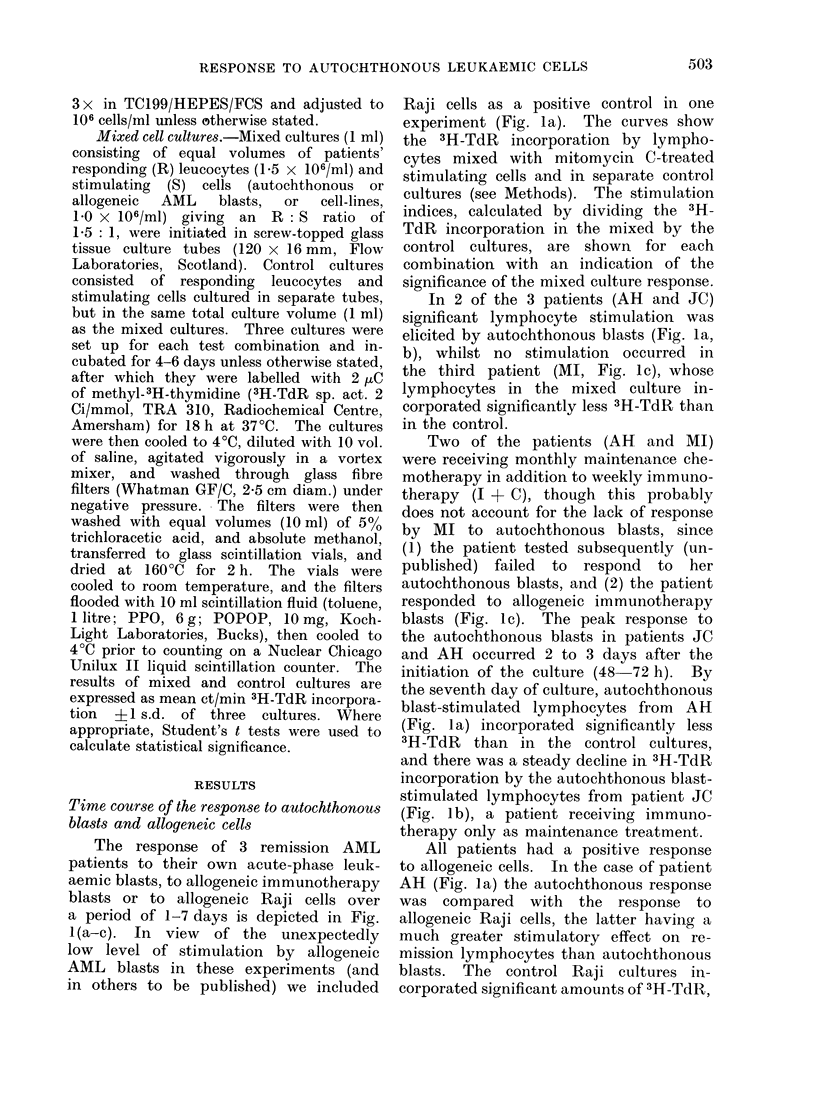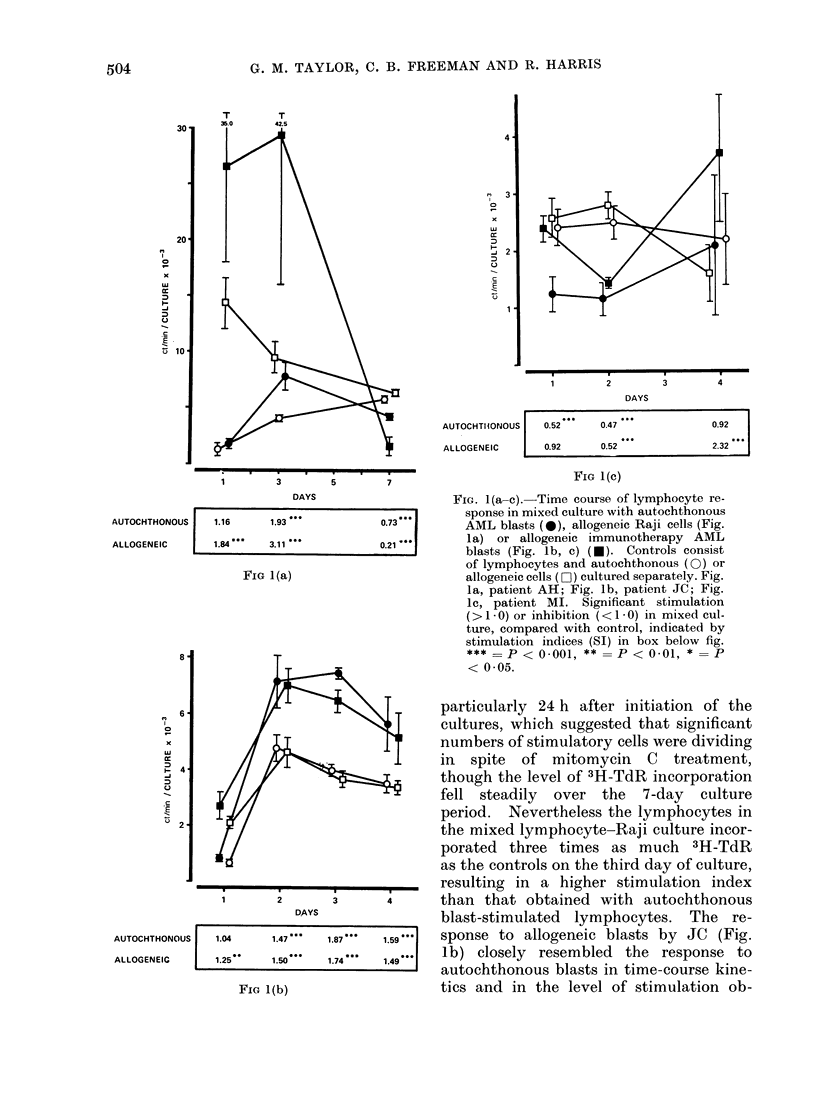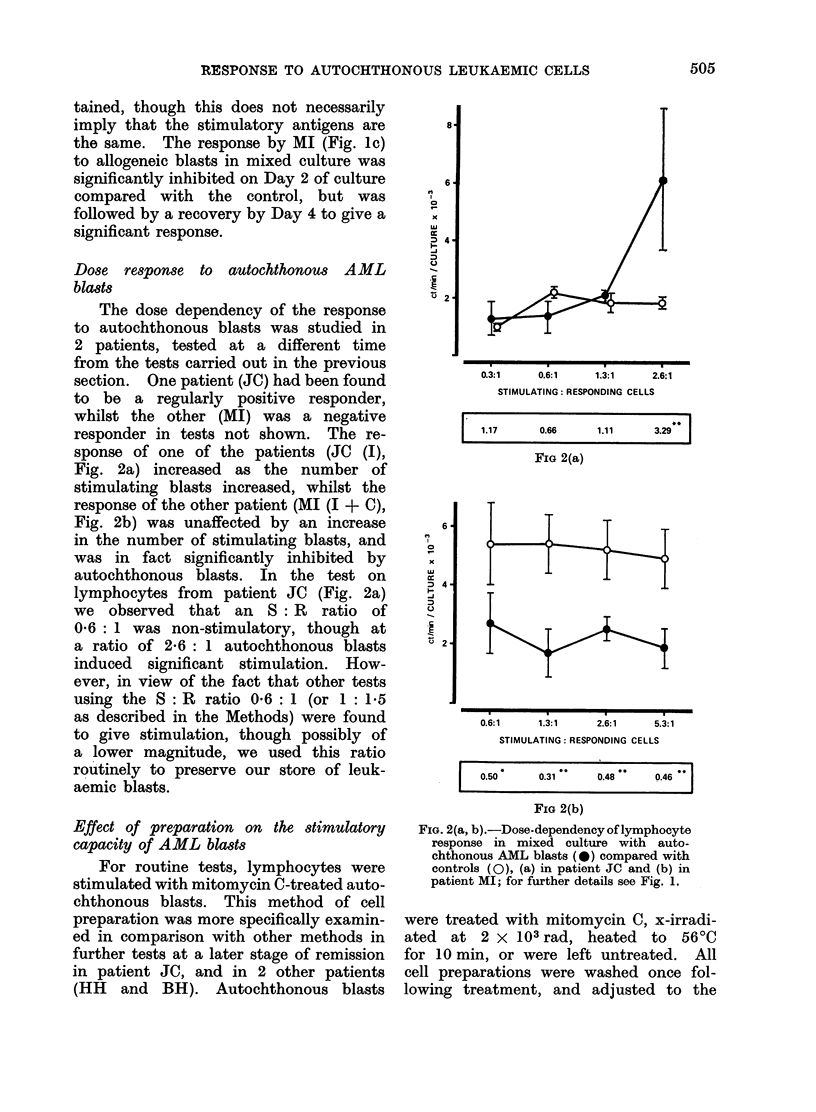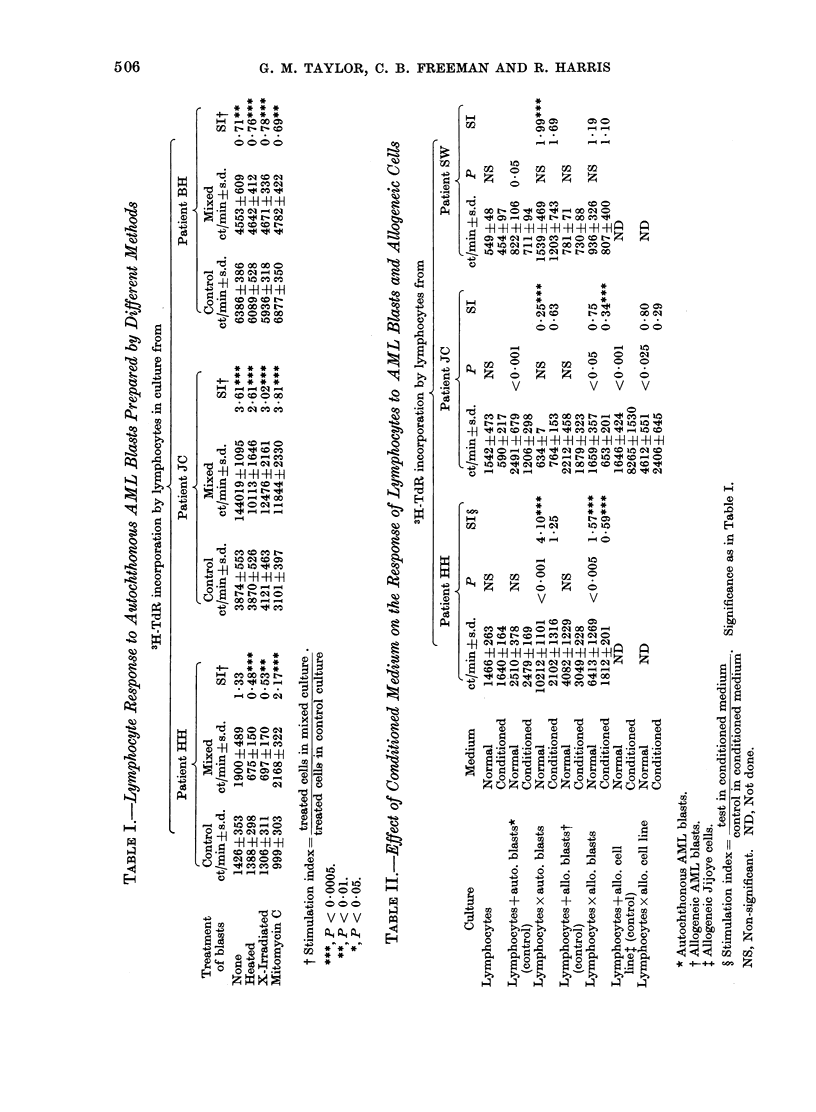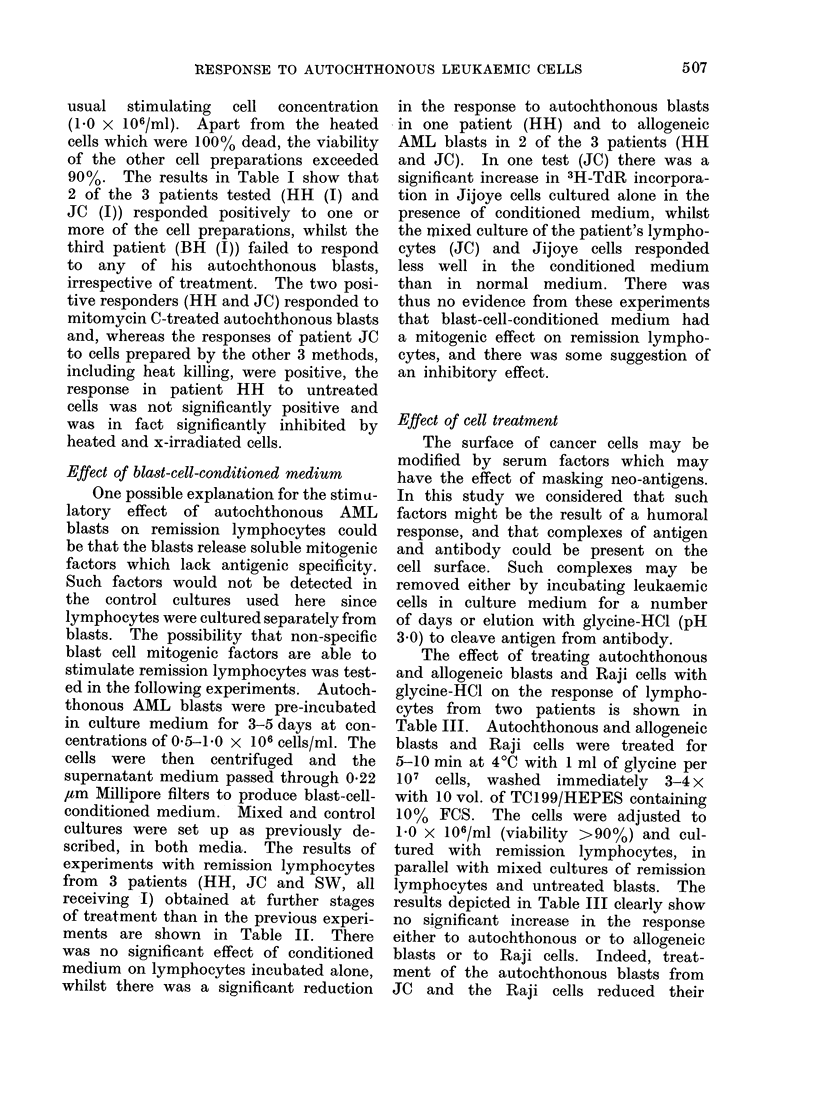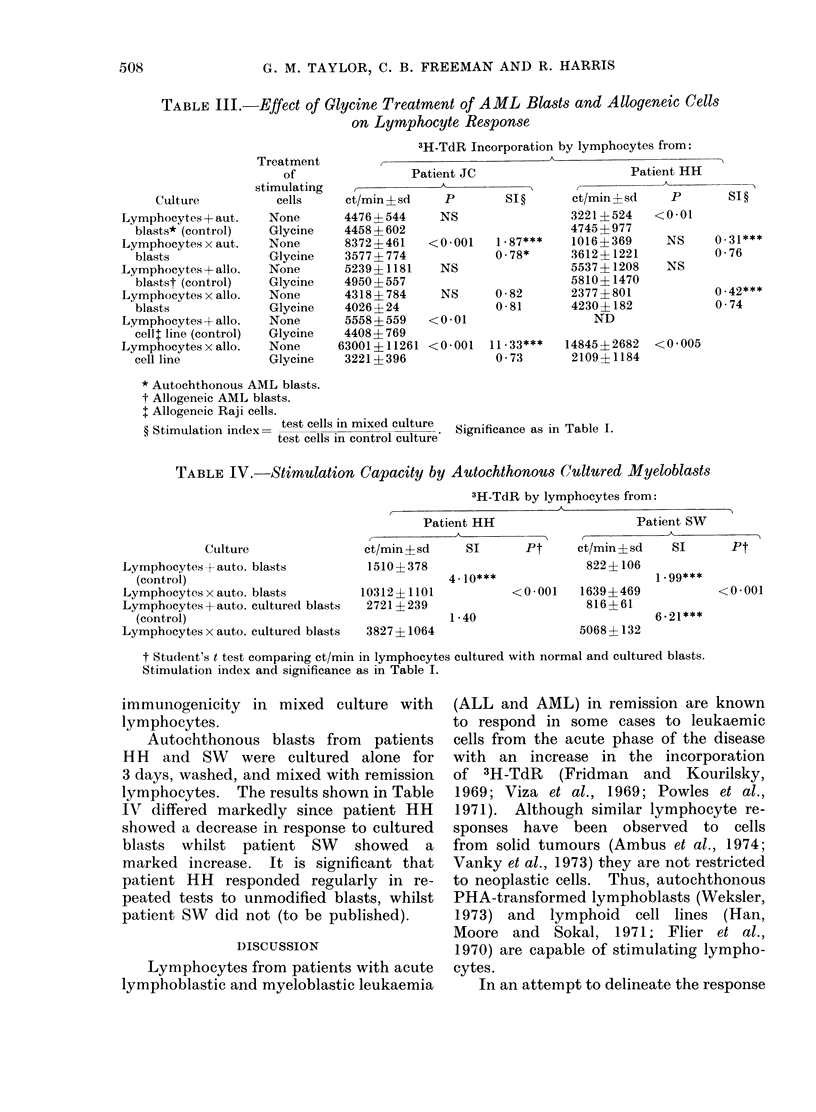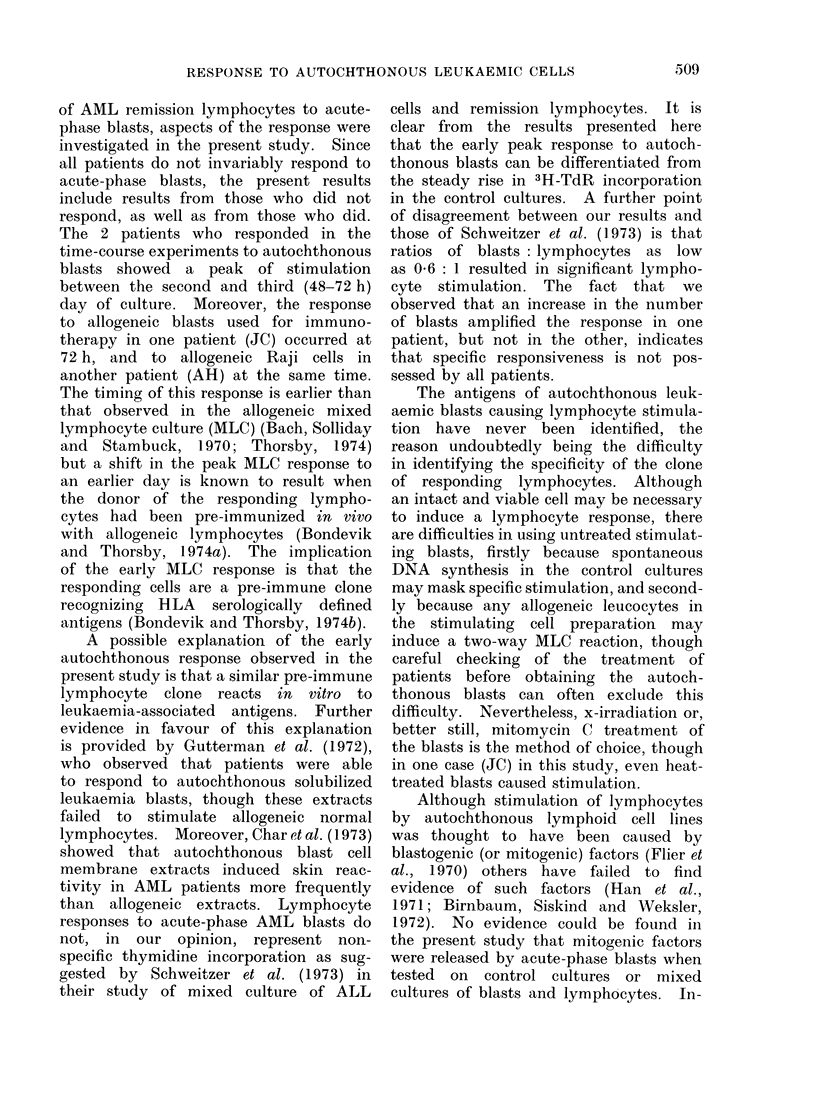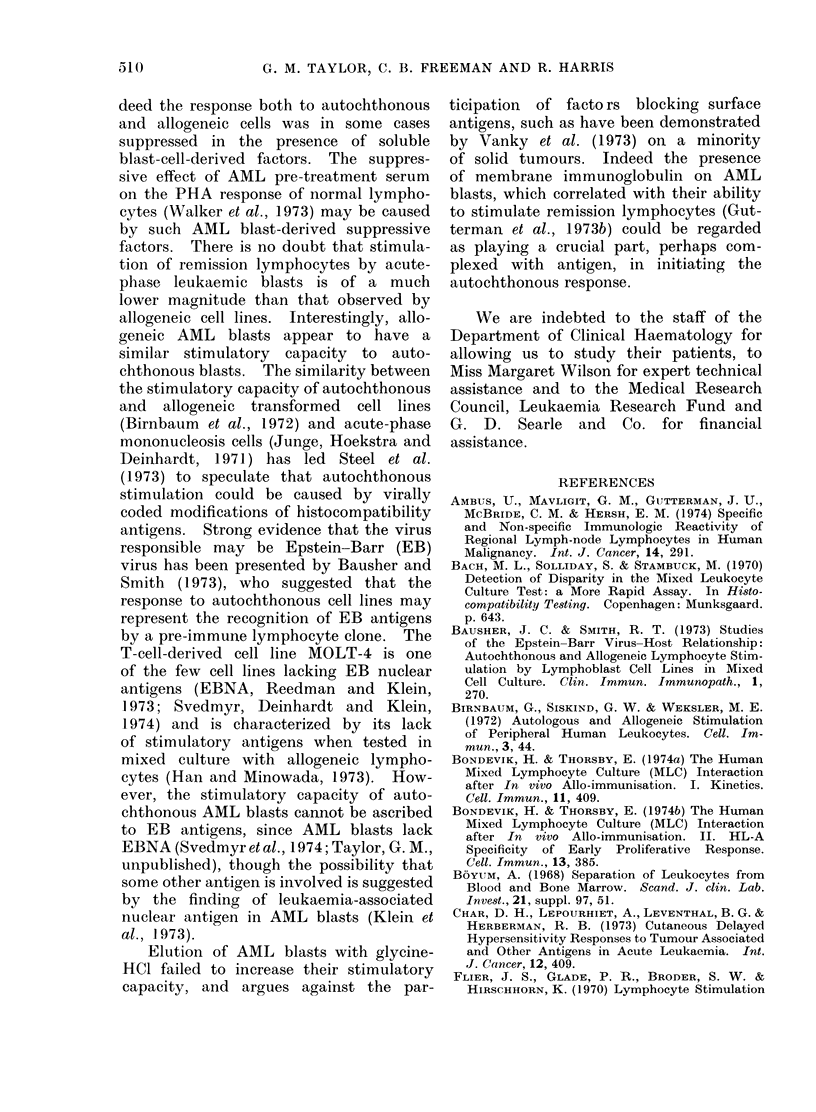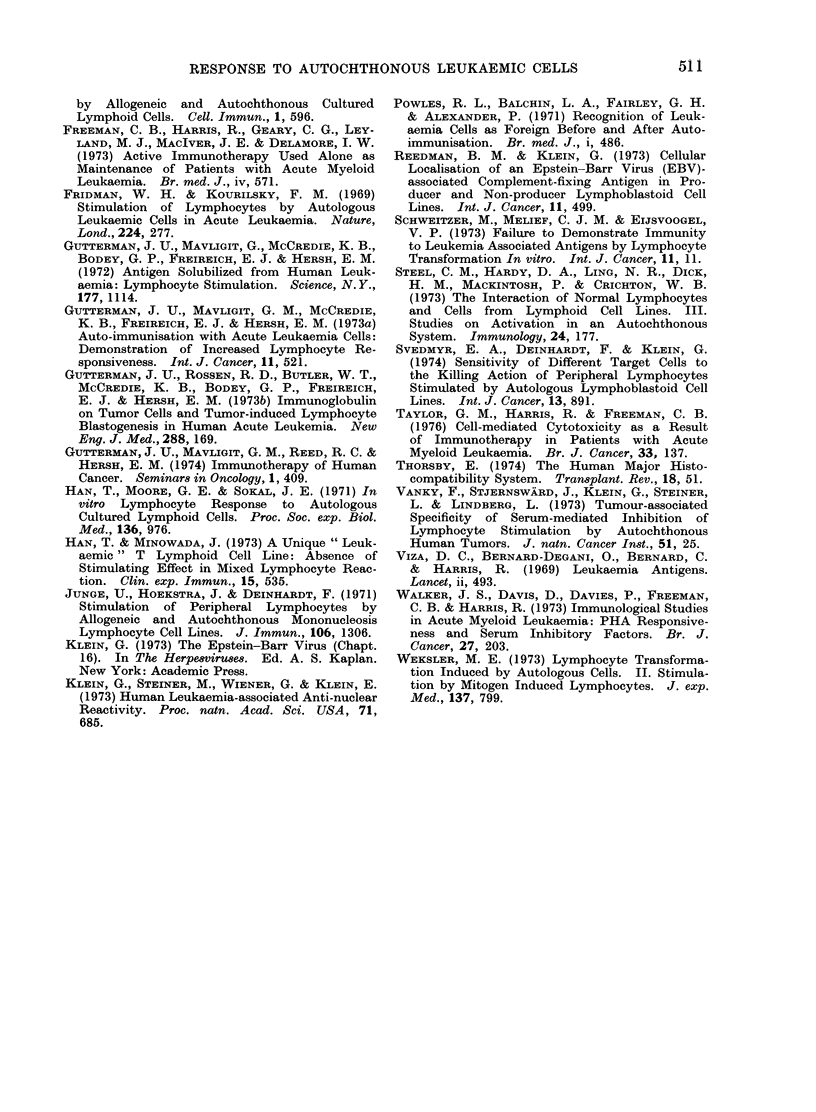# Response of remission lymphocytes to autochthonous leukaemic myeloblasts.

**DOI:** 10.1038/bjc.1976.81

**Published:** 1976-05

**Authors:** G. M. Taylor, C. B. Freeman, R. Harris

## Abstract

Thymidine incorporation in vitro by remission lymphocytes from a total of 6 patients with acute myeloid leukaemia (AML) was measured following stimulation by autochthonous and allogeneic AML blasts and cell lines. The early peak response to autochthonous blasts in 2 of these patients (48-72 h) is consistent with the concept of a population of lymphocytes pre-immunized to antigens carried by the blasts. Although stimulation in one patient was increased in the presence of more stimulating (S) blasts than responding (R) lymphocytes, positive responses in other tests were obtained at an S : R ratio of 1 : 1-5. When different methods of treatment of the stimulating autochthonous blasts were compared with untreated cells, mitomycin C gave the highest stimulation indices 2 out of 3 tests. Tissue culture medium in which autochthonous blasts had been incubated for 3-5 days failed to stimulate either remission lymphocytes alone, or combined cultures of lymphocytes with autochthonous or allogeneic blasts, suggesting that mitogenic factors released from autochthonous blasts are not responsible for lymphocyte stimulation. Treatment of autochthonous or allogeneic AML blasts with glycine-HC1(pH 3-0) to remove putative "blocking" factors failed to increase the stimulatory capacity of the leukaemic blasts.


Br. J. Cancer (1976) 33, 501

### RESPONSE OF REMISSION LYMPHOCYTES TO AUTOCHTHONOUS LEUKAEMIC MYELOBLASTS

G. M. TAYLOR, C. B. FREEMAN AND R. HARRIS

*From the University Department of Medical Genetics, St Mary's Hospital, Hathersage Road, Manchester, M13 0JH*

Received 3 October 1975 Accepted 7 January 1976

**Summary.—Thymidine incorporation *in vitro* by remission lymphocytes from a total of 6 patients with acute myeloid leukaemia (AML) was measured following stimulation by autochthonous and allogeneic AML blasts and cell lines. The early peak response to autochthonous blasts in 2 of these patients (48–72 h) is consistent with the concept of a population of lymphocytes pre-immunized to antigens carried by the blasts. Although stimulation in one patient was increased in the presence of more stimulating (S) blasts than responding (R) lymphocytes, positive responses in other tests were obtained at an S : R ratio of 1 : 1·5. When different methods of treatment of the stimulating autochthonous blasts were compared with untreated cells, mitomycin C gave the highest stimulation indices in 2 out of 3 tests. Tissue culture medium in which autochthonous blasts had been incubated for 3–5 days failed to stimulate either remission lymphocytes alone, or combined cultures of lymphocytes with autochthonous or allogeneic blasts, suggesting that mitogenic factors released from autochthonous blasts are not responsible for lymphocyte stimulation. Treatment of autochthonous or allogeneic AML blasts with glycine-HCl (pH 3·0) to remove putative " blocking " factors failed to increase the stimulatory capacity of the leukaemic blasts.**

THE SEARCH for antigens characteristic of neoplastic cells which may have diagnostic or therapeutic significance has been one of the primary aims of tumour immunology. The evidence of such antigens specific to human leukaemic cells is based largely on observations that DNA synthesis by lymphocytes from patients in remission is stimulated by autochthonous acute-phase leukaemic blasts (Fridman and Kourilsky, 1969; Viza *et al.*, 1969; Powles *et al.*, 1971) though the existence of such antigens on lymphoblastic leukaemic cells using this test has been disputed (Schweitzer, Melief and Eijsvoogel, 1973).

The clinical value of this test appears to be as an indicator of prognosis and minimal residual disease. Thus patients whose lymphocytes are strongly stimulated by autochthonous blasts are more likely to enter and remain in remission for more than one year, than those whose lymphocytes are weakly stimulated (Gutterman *et al.*, 1974).

In view of the potential clinical importance of the lymphocyte stimulation test in predicting remission length and residual disease, its possible use in monitoring the effects of immunotherapy (Powles *et al.*, 1971; Gutterman *et al.*, 1973*a*) and the induction of cell-mediated cytotoxicity (Taylor, Harris and Freeman, 1976), further clarification of its nature is long overdue. This study investigates variables determining the response to autochthonous AML blasts by lymphocytes from AML patients in remission who are receiving allogeneic AML blasts as part of their immunotherapy.

### MATERIALS AND METHODS

*Patients: remission induction.*—The 6 patients (JC, MI, AH, HH, SW and BH) whose responses to autochthonous leukaemic blasts and allogeneic cells were measured were all treated with induction chemotherapy consisting of cytosine arabinoside (Ara-C, 2·0 mg/kg body wt) administered i.v. in 5-day courses with gaps of 5 days, and daunorubicin (1·5 mg/kg body wt) in 6 courses coinciding with the first day of Ara-C. When the patients were in full remission (<5% blasts in the bone marrow) they received a single course of Ara-C/daunorubicin as above, and commenced immunotherapy one week later.

*Maintenance treatment.*—The patients were then randomized to receive weekly immunotherapy according to one of two protocols. Both groups received weekly injections of $10^9$ viable, allogeneic AML blasts pre-irradiated at $10^4$ rad ($^{137}Cs$ source), distributed between three limbs, and $10^9$ live BCG organisms (Glaxo Laboratories, Greenford) delivered into the fourth limb by multiple puncture with a Heaf gun (Freeman *et al.*, 1973). In addition, one of the groups of patients received monthly maintenance chemotherapy consisting of 5-day courses of Ara-C (2·0 mg/kg orally) and daunorubicin (1·5 mg/kg), the latter at 2-monthly intervals.

*Leukaemic blasts.*—Leukaemic blasts were obtained from the peripheral blood of the patients prior to induction chemotherapy, either by aspiration of venous blood into heparinized bottles or, where clinically indicated, by leucophoresis by means of an NCI–IBM continuous-flow cell separator. In the case of the leucophoresis patients, plasma and erythrocytes were returned to the patient, whilst the buffy coat was collected into a sterile blood bottle. Where whole venous blood was collected, erythrocytes were sedimented at 37 °C with Dextran 110 (Fisons, Loughborough).

Leucocytes obtained by leucophoresis and dextran sedimentation were diluted with antibiotic-free TC199, buffered with sodium bicarbonate or HEPES (Gibco Bio-Cult, Glasgow) containing dimethyl sulphoxide (DMSO) at 4 °C to give a final cell suspension containing 10% autologous plasma, 10% DMSO and 80% TC199. The cell suspensions were dispensed in 2 ml aliquots at concentrations of $10^7 - 2 \times 10^7$/ml into sterile 5 ml glass ampoules, which were then sealed and cooled to 4 °C. The ampoules were frozen at a rate of −1 °C/min to −150 °C in a programmed freezer (R201, G. V. Planer Ltd) and then stored at −120 °C in the vapour phase of a liquid $N_2$ refrigerator (LR-320, Union Carbide, Darlington).

*Preparation of responding lymphocytes.*—Venous blood obtained from remission AML patients was anticoagulated with heparin (10 iu/ml) or by defibrination with sterile glass beads. A lymphocyte-enriched leucocyte population was obtained by layering the whole blood—in some cases diluted 1 : 1 with sterile Ringer solution (Baxter Laboratories, Thetford, Norfolk)—over 5 ml (whole blood) or 3 ml (diluted blood) of a mixture of 9% Ficoll (Pharmacia, Uppsala, Sweden) and 33% Triosil (Nyegaard, Oslo, Norway) (24 parts: 10 parts) in straight-sided flat-bottomed glass tubes (100 × 14 mm) followed by centrifugation at 4 °C for 40 min at 400 *g* (Böyum, 1968). The interface of the plasma and Ficoll/Triosil-containing lymphocyte-enriched leucocytes was gently removed with glass pipettes, placed in glass centrifuge tubes and washed twice by centrifugation in TC199/HEPES containing 10% foetal calf serum (FCS) (Wellcome Laboratories, Kent). The cells were adjusted to $1·5 \times 10^6$/ml by counting in trypan blue.

*Preparation of stimulating cells.*—The autochthonous and allogeneic cells used as stimulating cells were obtained from storage in liquid $N_2$, and were thawed rapidly by agitation of the ampoules in a water bath at 37 °C, after which the cells were washed once in cold TC199/HEPES containing 10% FCS.

Allogeneic cell lines used as stimulating cells in some of the experiments were derived originally from Burkitt's lymphomata and designated Raji or Jijoye (see Klein, 1973). They were maintained in suspension culture in Falcon 3013 culture flasks (Falcon Plastics, Oxnard, California) in suspension Eagles Medium (S-MEM, Gibco Bio-Cult, Glasgow) containing 10% FCS and sub-cultured once or twice weekly.

All stimulating cells were treated with mitomycin C (25 $\mu g/10^7$ cells) unless otherwise stated, for 30 min at 37 °C to inhibit DNA synthesis by the stimulating cells whilst in mixed culture. The cells were then washed

3× in TC199/HEPES/FCS and adjusted to $10^6$ cells/ml unless otherwise stated.

*Mixed cell cultures.*—Mixed cultures (1 ml) consisting of equal volumes of patients' responding (R) leucocytes ($1\cdot5 \times 10^6$/ml) and stimulating (S) cells (autochthonous or allogeneic AML blasts, or cell-lines, $1\cdot0 \times 10^6$/ml) giving an R : S ratio of 1·5 : 1, were initiated in screw-topped glass tissue culture tubes (120 × 16 mm, Flow Laboratories, Scotland). Control cultures consisted of responding leucocytes and stimulating cells cultured in separate tubes, but in the same total culture volume (1 ml) as the mixed cultures. Three cultures were set up for each test combination and incubated for 4–6 days unless otherwise stated, after which they were labelled with 2 $\mu$C of methyl-$^3$H-thymidine ($^3$H-TdR sp. act. 2 Ci/mmol, TRA 310, Radiochemical Centre, Amersham) for 18 h at 37°C. The cultures were then cooled to 4°C, diluted with 10 vol. of saline, agitated vigorously in a vortex mixer, and washed through glass fibre filters (Whatman GF/C, 2·5 cm diam.) under negative pressure. The filters were then washed with equal volumes (10 ml) of 5% trichloracetic acid, and absolute methanol, transferred to glass scintillation vials, and dried at 160°C for 2 h. The vials were cooled to room temperature, and the filters flooded with 10 ml scintillation fluid (toluene, 1 litre; PPO, 6 g; POPOP, 10 mg, Koch-Light Laboratories, Bucks), then cooled to 4°C prior to counting on a Nuclear Chicago Unilux II liquid scintillation counter. The results of mixed and control cultures are expressed as mean ct/min $^3$H-TdR incorporation $\pm$1 s.d. of three cultures. Where appropriate, Student's $t$ tests were used to calculate statistical significance.

### RESULTS

### *Time course of the response to autochthonous blasts and allogeneic cells*

The response of 3 remission AML patients to their own acute-phase leukaemic blasts, to allogeneic immunotherapy blasts or to allogeneic Raji cells over a period of 1–7 days is depicted in Fig. 1(a–c). In view of the unexpectedly low level of stimulation by allogeneic AML blasts in these experiments (and in others to be published) we included Raji cells as a positive control in one experiment (Fig. 1a). The curves show the $^3$H-TdR incorporation by lymphocytes mixed with mitomycin C-treated stimulating cells and in separate control cultures (see Methods). The stimulation indices, calculated by dividing the $^3$H-TdR incorporation in the mixed by the control cultures, are shown for each combination with an indication of the significance of the mixed culture response.

In 2 of the 3 patients (AH and JC) significant lymphocyte stimulation was elicited by autochthonous blasts (Fig. 1a, b), whilst no stimulation occurred in the third patient (MI, Fig. 1c), whose lymphocytes in the mixed culture incorporated significantly less $^3$H-TdR than in the control.

Two of the patients (AH and MI) were receiving monthly maintenance chemotherapy in addition to weekly immunotherapy (I + C), though this probably does not account for the lack of response by MI to autochthonous blasts, since (1) the patient tested subsequently (unpublished) failed to respond to her autochthonous blasts, and (2) the patient responded to allogeneic immunotherapy blasts (Fig. 1c). The peak response to the autochthonous blasts in patients JC and AH occurred 2 to 3 days after the initiation of the culture (48—72 h). By the seventh day of culture, autochthonous blast-stimulated lymphocytes from AH (Fig. 1a) incorporated significantly less $^3$H-TdR than in the control cultures, and there was a steady decline in $^3$H-TdR incorporation by the autochthonous blast-stimulated lymphocytes from patient JC (Fig. 1b), a patient receiving immunotherapy only as maintenance treatment.

All patients had a positive response to allogeneic cells. In the case of patient AH (Fig. 1a) the autochthonous response was compared with the response to allogeneic Raji cells, the latter having a much greater stimulatory effect on remission lymphocytes than autochthonous blasts. The control Raji cultures incorporated significant amounts of $^3$H-TdR,

| | | | | |
|---|---|---|---|---|
| AUTOCHTHONOUS | 1.16 | 1.93*** | | 0.73*** |
| ALLOGENEIC | 1.84*** | 3.11*** | | 0.21*** |

FIG 1(a)

| | | | | |
|---|---|---|---|---|
| AUTOCHTHONOUS | 0.52*** | 0.47*** | | 0.92 |
| ALLOGENEIC | 0.92 | 0.52*** | | 2.32*** |

FIG 1(c)

FIG. 1(a–c).—Time course of lymphocyte response in mixed culture with autochthonous AML blasts (●), allogeneic Raji cells (Fig. 1a) or allogeneic immunotherapy AML blasts (Fig. 1b, c) (■). Controls consist of lymphocytes and autochthonous (○) or allogeneic cells (□) cultured separately. Fig. 1a, patient AH; Fig. 1b, patient JC; Fig. 1c, patient MI. Significant stimulation ($>1 \cdot 0$) or inhibition ($<1 \cdot 0$) in mixed culture, compared with control, indicated by stimulation indices (SI) in box below fig. *** $= P < 0 \cdot 001$, ** $= P < 0 \cdot 01$, * $= P < 0 \cdot 05$.

| | | | | |
|---|---|---|---|---|
| AUTOCHTHONOUS | 1.04 | 1.47*** | 1.87*** | 1.59*** |
| ALLOGENEIC | 1.25** | 1.50*** | 1.74*** | 1.49*** |

FIG 1(b)

particularly 24 h after initiation of the cultures, which suggested that significant numbers of stimulatory cells were dividing in spite of mitomycin C treatment, though the level of $^{3}$H-TdR incorporation fell steadily over the 7-day culture period. Nevertheless the lymphocytes in the mixed lymphocyte–Raji culture incorporated three times as much $^{3}$H-TdR as the controls on the third day of culture, resulting in a higher stimulation index than that obtained with autochthonous blast-stimulated lymphocytes. The response to allogeneic blasts by JC (Fig. 1b) closely resembled the response to autochthonous blasts in time-course kinetics and in the level of stimulation obtained, though this does not necessarily imply that the stimulatory antigens are the same. The response by MI (Fig. 1c) to allogeneic blasts in mixed culture was significantly inhibited on Day 2 of culture compared with the control, but was followed by a recovery by Day 4 to give a significant response.

### *Dose response to autochthonous AML blasts*

The dose dependency of the response to autochthonous blasts was studied in 2 patients, tested at a different time from the tests carried out in the previous section. One patient (JC) had been found to be a regularly positive responder, whilst the other (MI) was a negative responder in tests not shown. The response of one of the patients (JC (I), Fig. 2a) increased as the number of stimulating blasts increased, whilst the response of the other patient (MI (I + C), Fig. 2b) was unaffected by an increase in the number of stimulating blasts, and was in fact significantly inhibited by autochthonous blasts. In the test on lymphocytes from patient JC (Fig. 2a) we observed that an S : R ratio of 0·6 : 1 was non-stimulatory, though at a ratio of 2·6 : 1 autochthonous blasts induced significant stimulation. However, in view of the fact that other tests using the S : R ratio 0·6 : 1 (or 1 : 1·5 as described in the Methods) were found to give stimulation, though possibly of a lower magnitude, we used this ratio routinely to preserve our store of leukaemic blasts.

FIG 2(a)

FIG 2(b)

FIG. 2(a, b).—Dose-dependency of lymphocyte response in mixed culture with autochthonous AML blasts (●) compared with controls (○), (a) in patient JC and (b) in patient MI; for further details see Fig. 1.

### *Effect of preparation on the stimulatory capacity of AML blasts*

For routine tests, lymphocytes were stimulated with mitomycin C-treated autochthonous blasts. This method of cell preparation was more specifically examined in comparison with other methods in further tests at a later stage of remission in patient JC, and in 2 other patients (HH and BH). Autochthonous blasts were treated with mitomycin C, x-irradiated at $2 \times 10^3$ rad, heated to 56°C for 10 min, or were left untreated. All cell preparations were washed once following treatment, and adjusted to the

TABLE I.—*Lymphocyte Response to Autochthonous AML Blasts Prepared by Different Methods*

| Treatment of blasts | $^3$H-TdR incorporation by lymphocytes in culture from | | | | | | | | |
|---|---|---|---|---|---|---|---|---|---|
| | Patient HH | | | Patient JC | | | Patient BH | | |
| | Control ct/min ± s.d. | Mixed ct/min ± s.d. | SI† | Control ct/min ± s.d. | Mixed ct/min ± s.d. | SI† | Control ct/min ± s.d. | Mixed ct/min ± s.d. | SI† |
| None | 1426 ± 353 | 1900 ± 489 | 1·33 | 3874 ± 553 | 144019 ± 1095 | 3·61*** | 6386 ± 386 | 4553 ± 609 | 0·71** |
| Heated | 1388 ± 298 | 675 ± 150 | 0·48*** | 3870 ± 526 | 10113 ± 1646 | 2·61*** | 6089 ± 528 | 4642 ± 412 | 0·76*** |
| X-Irradiated | 1306 ± 311 | 697 ± 170 | 0·53** | 4121 ± 463 | 12476 ± 2161 | 3·02*** | 5936 ± 318 | 4671 ± 336 | 0·78*** |
| Mitomycin C | 999 ± 303 | 2168 ± 322 | 2·17*** | 3101 ± 397 | 11844 ± 2330 | 3·81*** | 6877 ± 350 | 4782 ± 422 | 0·69** |

† Stimulation index = $\frac{\text{treated cells in mixed culture}}{\text{treated cells in control culture}}$.

***, $P < 0·0005$.

**, $P < 0·01$.

*, $P < 0·05$.

TABLE II.—*Effect of Conditioned Medium on the Response of Lymphocytes to AML Blasts and Allogeneic Cells*

| Culture | Medium | $^3$H-TdR incorporation by lymphocytes from | | | | | | | | |
|---|---|---|---|---|---|---|---|---|---|---|
| | | Patient HH | | | Patient JC | | | Patient SW | | |
| | | ct/min ± s.d. | $P$ | SI§ | ct/min ± s.d. | $P$ | SI | ct/min ± s.d. | $P$ | SI |
| Lymphocytes | Normal | 1466 ± 263 | NS | | 1542 ± 473 | NS | | 549 ± 48 | NS | |
| | Conditioned | 1640 ± 164 | | | 590 ± 217 | | | 454 ± 97 | | |
| Lymphocytes + auto. blasts* (control) | Normal | 2510 ± 378 | NS | | 2491 ± 679 | < 0·001 | | 822 ± 106 | 0·05 | |
| | Conditioned | 2479 ± 169 | | | 1206 ± 298 | | | 711 ± 94 | | |
| Lymphocytes × auto. blasts | Normal | 10212 ± 1101 | < 0·001 | 4·10*** | 634 ± 7 | NS | 0·25*** | 1539 ± 469 | NS | 1·99*** |
| | Conditioned | 2102 ± 1316 | | 1·25 | 764 ± 153 | | 0·63 | 1203 ± 743 | | 1·69 |
| Lymphocytes + allo. blasts† (control) | Normal | 4082 ± 1229 | NS | | 2212 ± 458 | NS | | 781 ± 71 | NS | |
| | Conditioned | 3049 ± 228 | | | 1879 ± 323 | | | 730 ± 88 | | |
| Lymphocytes × allo. blasts | Normal | 6413 ± 1269 | < 0·005 | 1·57*** | 1659 ± 357 | < 0·05 | 0·75 | 936 ± 326 | NS | 1·19 |
| | Conditioned | 1812 ± 201 | | 0·59*** | 653 ± 201 | | 0·34*** | 807 ± 400 | | 1·10 |
| Lymphocytes + allo. cell line‡ (control) | Normal | ND | | | 1646 ± 424 | < 0·001 | | ND | | |
| | Conditioned | | | | 8265 ± 1530 | | | | | |
| Lymphocytes × allo. cell line | Normal | ND | | | 4612 ± 551 | < 0·025 | 0·80 | ND | | |
| | Conditioned | | | | 2406 ± 645 | | 0·29 | | | |

* Autochthonous AML blasts.

† Allogeneic AML blasts.

‡ Allogeneic Jijoye cells.

§ Stimulation index = $\frac{\text{test in conditioned medium}}{\text{control in conditioned medium}}$. Significance as in Table I.

NS, Non-significant. ND, Not done.

usual stimulating cell concentration ($1 \cdot 0 \times 10^6$/ml). Apart from the heated cells which were 100% dead, the viability of the other cell preparations exceeded 90%. The results in Table I show that 2 of the 3 patients tested (HH (I) and JC (I)) responded positively to one or more of the cell preparations, whilst the third patient (BH (I)) failed to respond to any of his autochthonous blasts, irrespective of treatment. The two positive responders (HH and JC) responded to mitomycin C-treated autochthonous blasts and, whereas the responses of patient JC to cells prepared by the other 3 methods, including heat killing, were positive, the response in patient HH to untreated cells was not significantly positive and was in fact significantly inhibited by heated and x-irradiated cells.

*Effect of blast-cell-conditioned medium*

One possible explanation for the stimulatory effect of autochthonous AML blasts on remission lymphocytes could be that the blasts release soluble mitogenic factors which lack antigenic specificity. Such factors would not be detected in the control cultures used here since lymphocytes were cultured separately from blasts. The possibility that non-specific blast cell mitogenic factors are able to stimulate remission lymphocytes was tested in the following experiments. Autochthonous AML blasts were pre-incubated in culture medium for 3–5 days at concentrations of $0 \cdot 5$–$1 \cdot 0 \times 10^6$ cells/ml. The cells were then centrifuged and the supernatant medium passed through 0·22 μm Millipore filters to produce blast-cell-conditioned medium. Mixed and control cultures were set up as previously described, in both media. The results of experiments with remission lymphocytes from 3 patients (HH, JC and SW, all receiving I) obtained at further stages of treatment than in the previous experiments are shown in Table II. There was no significant effect of conditioned medium on lymphocytes incubated alone, whilst there was a significant reduction in the response to autochthonous blasts in one patient (HH) and to allogeneic AML blasts in 2 of the 3 patients (HH and JC). In one test (JC) there was a significant increase in $^3$H-TdR incorporation in Jijoye cells cultured alone in the presence of conditioned medium, whilst the mixed culture of the patient's lymphocytes (JC) and Jijoye cells responded less well in the conditioned medium than in normal medium. There was thus no evidence from these experiments that blast-cell-conditioned medium had a mitogenic effect on remission lymphocytes, and there was some suggestion of an inhibitory effect.

*Effect of cell treatment*

The surface of cancer cells may be modified by serum factors which may have the effect of masking neo-antigens. In this study we considered that such factors might be the result of a humoral response, and that complexes of antigen and antibody could be present on the cell surface. Such complexes may be removed either by incubating leukaemic cells in culture medium for a number of days or elution with glycine-HCl (pH 3·0) to cleave antigen from antibody.

The effect of treating autochthonous and allogeneic blasts and Raji cells with glycine-HCl on the response of lymphocytes from two patients is shown in Table III. Autochthonous and allogeneic blasts and Raji cells were treated for 5–10 min at 4°C with 1 ml of glycine per $10^7$ cells, washed immediately 3–4× with 10 vol. of TC199/HEPES containing 10% FCS. The cells were adjusted to $1 \cdot 0 \times 10^6$/ml (viability >90%) and cultured with remission lymphocytes, in parallel with mixed cultures of remission lymphocytes and untreated blasts. The results depicted in Table III clearly show no significant increase in the response either to autochthonous or to allogeneic blasts or to Raji cells. Indeed, treatment of the autochthonous blasts from JC and the Raji cells reduced their

TABLE III.—*Effect of Glycine Treatment of AML Blasts and Allogeneic Cells on Lymphocyte Response*

| Culture | Treatment of stimulating cells | $^3$H-TdR Incorporation by lymphocytes from: Patient JC ct/min ± sd | *P* | SI§ | Patient HH ct/min ± sd | *P* | SI§ |
|---|---|---|---|---|---|---|---|
| Lymphocytes + aut. | None | 4476 ± 544 | NS | | 3221 ± 524 | <0·01 | |
| blasts* (control) | Glycine | 4458 ± 602 | | | 4745 ± 977 | | |
| Lymphocytes × aut. | None | 8372 ± 461 | <0·001 | 1·87*** | 1016 ± 369 | NS | 0·31*** |
| blasts | Glycine | 3577 ± 774 | | 0·78* | 3612 ± 1221 | | 0·76 |
| Lymphocytes + allo. | None | 5239 ± 1181 | NS | | 5537 ± 1208 | NS | |
| blasts† (control) | Glycine | 4950 ± 557 | | | 5810 ± 1470 | | |
| Lymphocytes × allo. | None | 4318 ± 784 | NS | 0·82 | 2377 ± 801 | | 0·42*** |
| blasts | Glycine | 4026 ± 24 | | 0·81 | 4230 ± 182 | | 0·74 |
| Lymphocytes + allo. | None | 5558 ± 559 | <0·01 | | ND | | |
| cell‡ line (control) | Glycine | 4408 ± 769 | | | | | |
| Lymphocytes × allo. | None | 63001 ± 11261 | <0·001 | 11·33*** | 14845 ± 2682 | <0·005 | |
| cell line | Glycine | 3221 ± 396 | | 0·73 | 2109 ± 1184 | | |

* Autochthonous AML blasts.
† Allogeneic AML blasts.
‡ Allogeneic Raji cells.
§ Stimulation index = $\frac{\text{test cells in mixed culture}}{\text{test cells in control culture}}$. Significance as in Table I.

TABLE IV.—*Stimulation Capacity by Autochthonous Cultured Myeloblasts*

| Culture | $^3$H-TdR by lymphocytes from: Patient HH ct/min ± sd | SI | *P*† | Patient SW ct/min ± sd | SI | *P*† |
|---|---|---|---|---|---|---|
| Lymphocytes + auto. blasts | 1510 ± 378 | | | 822 ± 106 | | |
| (control) | | 4·10*** | | | 1·99*** | |
| Lymphocytes × auto. blasts | 10312 ± 1101 | | <0·001 | 1639 ± 469 | | <0·001 |
| Lymphocytes + auto. cultured blasts | 2721 ± 239 | | | 816 ± 61 | | |
| (control) | | 1·40 | | | 6·21*** | |
| Lymphocytes × auto. cultured blasts | 3827 ± 1064 | | | 5068 ± 132 | | |

† Student's *t* test comparing ct/min in lymphocytes cultured with normal and cultured blasts. Stimulation index and significance as in Table I.

immunogenicity in mixed culture with lymphocytes.

Autochthonous blasts from patients HH and SW were cultured alone for 3 days, washed, and mixed with remission lymphocytes. The results shown in Table IV differed markedly since patient HH showed a decrease in response to cultured blasts whilst patient SW showed a marked increase. It is significant that patient HH responded regularly in repeated tests to unmodified blasts, whilst patient SW did not (to be published).

### DISCUSSION

Lymphocytes from patients with acute lymphoblastic and myeloblastic leukaemia (ALL and AML) in remission are known to respond in some cases to leukaemic cells from the acute phase of the disease with an increase in the incorporation of $^3$H-TdR (Fridman and Kourilsky, 1969; Viza *et al.*, 1969; Powles *et al.*, 1971). Although similar lymphocyte responses have been observed to cells from solid tumours (Ambus *et al.*, 1974; Vanky *et al.*, 1973) they are not restricted to neoplastic cells. Thus, autochthonous PHA-transformed lymphoblasts (Weksler, 1973) and lymphoid cell lines (Han, Moore and Sokal, 1971; Flier *et al.*, 1970) are capable of stimulating lymphocytes.

In an attempt to delineate the response of AML remission lymphocytes to acute-phase blasts, aspects of the response were investigated in the present study. Since all patients do not invariably respond to acute-phase blasts, the present results include results from those who did not respond, as well as from those who did. The 2 patients who responded in the time-course experiments to autochthonous blasts showed a peak of stimulation between the second and third (48–72 h) day of culture. Moreover, the response to allogeneic blasts used for immunotherapy in one patient (JC) occurred at 72 h, and to allogeneic Raji cells in another patient (AH) at the same time. The timing of this response is earlier than that observed in the allogeneic mixed lymphocyte culture (MLC) (Bach, Solliday and Stambuck, 1970; Thorsby, 1974) but a shift in the peak MLC response to an earlier day is known to result when the donor of the responding lymphocytes had been pre-immunized *in vivo* with allogeneic lymphocytes (Bondevik and Thorsby, 1974*a*). The implication of the early MLC response is that the responding cells are a pre-immune clone recognizing HLA serologically defined antigens (Bondevik and Thorsby, 1974*b*).

A possible explanation of the early autochthonous response observed in the present study is that a similar pre-immune lymphocyte clone reacts *in vitro* to leukaemia-associated antigens. Further evidence in favour of this explanation is provided by Gutterman *et al.* (1972), who observed that patients were able to respond to autochthonous solubilized leukaemia blasts, though these extracts failed to stimulate allogeneic normal lymphocytes. Moreover, Char *et al.* (1973) showed that autochthonous blast cell membrane extracts induced skin reactivity in AML patients more frequently than allogeneic extracts. Lymphocyte responses to acute-phase AML blasts do not, in our opinion, represent non-specific thymidine incorporation as suggested by Schweitzer *et al.* (1973) in their study of mixed culture of ALL cells and remission lymphocytes. It is clear from the results presented here that the early peak response to autochthonous blasts can be differentiated from the steady rise in $^3$H-TdR incorporation in the control cultures. A further point of disagreement between our results and those of Schweitzer *et al.* (1973) is that ratios of blasts : lymphocytes as low as 0·6 : 1 resulted in significant lymphocyte stimulation. The fact that we observed that an increase in the number of blasts amplified the response in one patient, but not in the other, indicates that specific responsiveness is not possessed by all patients.

The antigens of autochthonous leukaemic blasts causing lymphocyte stimulation have never been identified, the reason undoubtedly being the difficulty in identifying the specificity of the clone of responding lymphocytes. Although an intact and viable cell may be necessary to induce a lymphocyte response, there are difficulties in using untreated stimulating blasts, firstly because spontaneous DNA synthesis in the control cultures may mask specific stimulation, and secondly because any allogeneic leucocytes in the stimulating cell preparation may induce a two-way MLC reaction, though careful checking of the treatment of patients before obtaining the autochthonous blasts can often exclude this difficulty. Nevertheless, x-irradiation or, better still, mitomycin C treatment of the blasts is the method of choice, though in one case (JC) in this study, even heat-treated blasts caused stimulation.

Although stimulation of lymphocytes by autochthonous lymphoid cell lines was thought to have been caused by blastogenic (or mitogenic) factors (Flier *et al.*, 1970) others have failed to find evidence of such factors (Han *et al.*, 1971; Birnbaum, Siskind and Weksler, 1972). No evidence could be found in the present study that mitogenic factors were released by acute-phase blasts when tested on control cultures or mixed cultures of blasts and lymphocytes. Indeed the response both to autochthonous and allogeneic cells was in some cases suppressed in the presence of soluble blast-cell-derived factors. The suppressive effect of AML pre-treatment serum on the PHA response of normal lymphocytes (Walker *et al.*, 1973) may be caused by such AML blast-derived suppressive factors. There is no doubt that stimulation of remission lymphocytes by acute-phase leukaemic blasts is of a much lower magnitude than that observed by allogeneic cell lines. Interestingly, allogeneic AML blasts appear to have a similar stimulatory capacity to autochthonous blasts. The similarity between the stimulatory capacity of autochthonous and allogeneic transformed cell lines (Birnbaum *et al.*, 1972) and acute-phase mononucleosis cells (Junge, Hoekstra and Deinhardt, 1971) has led Steel *et al.* (1973) to speculate that autochthonous stimulation could be caused by virally coded modifications of histocompatibility antigens. Strong evidence that the virus responsible may be Epstein–Barr (EB) virus has been presented by Bausher and Smith (1973), who suggested that the response to autochthonous cell lines may represent the recognition of EB antigens by a pre-immune lymphocyte clone. The T-cell-derived cell line MOLT-4 is one of the few cell lines lacking EB nuclear antigens (EBNA, Reedman and Klein, 1973; Svedmyr, Deinhardt and Klein, 1974) and is characterized by its lack of stimulatory antigens when tested in mixed culture with allogeneic lymphocytes (Han and Minowada, 1973). However, the stimulatory capacity of autochthonous AML blasts cannot be ascribed to EB antigens, since AML blasts lack EBNA (Svedmyr *et al.*, 1974; Taylor, G. M., unpublished), though the possibility that some other antigen is involved is suggested by the finding of leukaemia-associated nuclear antigen in AML blasts (Klein *et al.*, 1973).

Elution of AML blasts with glycine-HCl failed to increase their stimulatory capacity, and argues against the participation of factors blocking surface antigens, such as have been demonstrated by Vanky *et al.* (1973) on a minority of solid tumours. Indeed the presence of membrane immunoglobulin on AML blasts, which correlated with their ability to stimulate remission lymphocytes (Gutterman *et al.*, 1973*b*) could be regarded as playing a crucial part, perhaps complexed with antigen, in initiating the autochthonous response.

We are indebted to the staff of the Department of Clinical Haematology for allowing us to study their patients, to Miss Margaret Wilson for expert technical assistance and to the Medical Research Council, Leukaemia Research Fund and G. D. Searle and Co. for financial assistance.

### REFERENCES

AMBUS, U., MAVLIGIT, G. M., GUTTERMAN, J. U., MCBRIDE, C. M. & HERSH, E. M. (1974) Specific and Non-specific Immunologic Reactivity of Regional Lymph-node Lymphocytes in Human Malignancy. *Int. J. Cancer*, **14**, 291.

BACH, M. L., SOLLIDAY, S. & STAMBUCK, M. (1970) Detection of Disparity in the Mixed Leukocyte Culture Test: a More Rapid Assay. In *Histocompatibility Testing*. Copenhagen: Munksgaard. p. 643.

BAUSHER, J. C. & SMITH, R. T. (1973) Studies of the Epstein–Barr Virus–Host Relationship: Autochthonous and Allogeneic Lymphocyte Stimulation by Lymphoblast Cell Lines in Mixed Cell Culture. *Clin. Immun. Immunopath.*, **1**, 270.

BIRNBAUM, G., SISKIND, G. W. & WEKSLER, M. E. (1972) Autologous and Allogeneic Stimulation of Peripheral Human Leukocytes. *Cell. Immun.*, **3**, 44.

BONDEVIK, H. & THORSBY, E. (1974*a*) The Human Mixed Lymphocyte Culture (MLC) Interaction after *In vivo* Allo-immunisation. I. Kinetics. *Cell. Immun.*, **11**, 409.

BONDEVIK, H. & THORSBY, E. (1974*b*) The Human Mixed Lymphocyte Culture (MLC) Interaction after *In vivo* Allo-immunisation. II. HL-A Specificity of Early Proliferative Response. *Cell. Immun.*, **13**, 385.

BÖYUM, A. (1968) Separation of Leukocytes from Blood and Bone Marrow. *Scand. J. clin. Lab. Invest.*, **21**, suppl. 97, 51.

CHAR, D. H., LEPOURHIET, A., LEVENTHAL, B. G. & HERBERMAN, R. B. (1973) Cutaneous Delayed Hypersensitivity Responses to Tumour Associated and Other Antigens in Acute Leukaemia. *Int. J. Cancer*, **12**, 409.

FLIER, J. S., GLADE, P. R., BRODER, S. W. & HIRSCHHORN, K. (1970) Lymphocyte Stimulation by Allogeneic and Autochthonous Cultured Lymphoid Cells. *Cell. Immun.*, **1**, 596.

FREEMAN, C. B., HARRIS, R., GEARY, C. G., LEYLAND, M. J., MACIVER, J. E. & DELAMORE, I. W. (1973) Active Immunotherapy Used Alone as Maintenance of Patients with Acute Myeloid Leukaemia. *Br. med. J.*, iv, 571.

FRIDMAN, W. H. & KOURILSKY, F. M. (1969) Stimulation of Lymphocytes by Autologous Leukaemic Cells in Acute Leukaemia. *Nature, Lond.*, **224**, 277.

GUTTERMAN, J. U., MAVLIGIT, G., MCCREDIE, K. B., BODEY, G. P., FREIREICH, E. J. & HERSH, E. M. (1972) Antigen Solubilized from Human Leukaemia: Lymphocyte Stimulation. *Science, N.Y.*, **177**, 1114.

GUTTERMAN, J. U., MAVLIGIT, G. M., MCCREDIE, K. B., FREIREICH, E. J. & HERSH, E. M. (1973*a*) Auto-immunisation with Acute Leukaemia Cells: Demonstration of Increased Lymphocyte Responsiveness. *Int. J. Cancer*, **11**, 521.

GUTTERMAN, J. U., ROSSEN, R. D., BUTLER, W. T., MCCREDIE, K. B., BODEY, G. P., FREIREICH, E. J. & HERSH, E. M. (1973*b*) Immunoglobulin on Tumor Cells and Tumor-induced Lymphocyte Blastogenesis in Human Acute Leukemia. *New Eng. J. Med.*, **288**, 169.

GUTTERMAN, J. U., MAVLIGIT, G. M., REED, R. C. & HERSH, E. M. (1974) Immunotherapy of Human Cancer. *Seminars in Oncology*, **1**, 409.

HAN, T., MOORE, G. E. & SOKAL, J. E. (1971) *In vitro* Lymphocyte Response to Autologous Cultured Lymphoid Cells. *Proc. Soc. exp. Biol. Med.*, **136**, 976.

HAN, T. & MINOWADA, J. (1973) A Unique " Leukaemic " T Lymphoid Cell Line: Absence of Stimulating Effect in Mixed Lymphocyte Reaction. *Clin. exp. Immun.*, **15**, 535.

JUNGE, U., HOEKSTRA, J. & DEINHARDT, F. (1971) Stimulation of Peripheral Lymphocytes by Allogeneic and Autochthonous Mononucleosis Lymphocyte Cell Lines. *J. Immun.*, **106**, 1306.

KLEIN, G. (1973) The Epstein–Barr Virus (Chapt. 16). In *The Herpesviruses*. Ed. A. S. Kaplan. New York: Academic Press.

KLEIN, G., STEINER, M., WIENER, G. & KLEIN, E. (1973) Human Leukaemia-associated Anti-nuclear Reactivity. *Proc. natn. Acad. Sci. USA*, **71**, 685.

POWLES, R. L., BALCHIN, L. A., FAIRLEY, G. H. & ALEXANDER, P. (1971) Recognition of Leukaemia Cells as Foreign Before and After Autoimmunisation. *Br. med. J.*, i, 486.

REEDMAN, B. M. & KLEIN, G. (1973) Cellular Localisation of an Epstein–Barr Virus (EBV)-associated Complement-fixing Antigen in Producer and Non-producer Lymphoblastoid Cell Lines. *Int. J. Cancer*, **11**, 499.

SCHWEITZER, M., MELIEF, C. J. M. & EIJSVOOGEL, V. P. (1973) Failure to Demonstrate Immunity to Leukemia Associated Antigens by Lymphocyte Transformation *In vitro*. *Int. J. Cancer*, **11**, 11.

STEEL, C. M., HARDY, D. A., LING, N. R., DICK, H. M., MACKINTOSH, P. & CRICHTON, W. B. (1973) The Interaction of Normal Lymphocytes and Cells from Lymphoid Cell Lines. III. Studies on Activation in an Autochthonous System. *Immunology*, **24**, 177.

SVEDMYR, E. A., DEINHARDT, F. & KLEIN, G. (1974) Sensitivity of Different Target Cells to the Killing Action of Peripheral Lymphocytes Stimulated by Autologous Lymphoblastoid Cell Lines. *Int. J. Cancer*, **13**, 891.

TAYLOR, G. M., HARRIS, R. & FREEMAN, C. B. (1976) Cell-mediated Cytotoxicity as a Result of Immunotherapy in Patients with Acute Myeloid Leukaemia. *Br. J. Cancer*, **33**, 137.

THORSBY, E. (1974) The Human Major Histocompatibility System. *Transplant. Rev.*, **18**, 51.

VANKY, F., STJERNSWÄRD, J., KLEIN, G., STEINER, L. & LINDBERG, L. (1973) Tumour-associated Specificity of Serum-mediated Inhibition of Lymphocyte Stimulation by Autochthonous Human Tumors. *J. natn. Cancer Inst.*, **51**, 25.

VIZA, D. C., BERNARD-DEGANI, O., BERNARD, C. & HARRIS, R. (1969) Leukaemia Antigens. *Lancet*, ii, 493.

WALKER, J. S., DAVIS, D., DAVIES, P., FREEMAN, C. B. & HARRIS, R. (1973) Immunological Studies in Acute Myeloid Leukaemia: PHA Responsiveness and Serum Inhibitory Factors. *Br. J. Cancer*, **27**, 203.

WEKSLER, M. E. (1973) Lymphocyte Transformation Induced by Autologous Cells. II. Stimulation by Mitogen Induced Lymphocytes. *J. exp. Med.*, **137**, 799.